# Goldilocks and the Three Bears: A Just-Right Hybrid Model to Synthesize the Growing Landscape of Publicly Available Health-Related Mobile Apps

**DOI:** 10.2196/27105

**Published:** 2021-06-07

**Authors:** Nancy Lau, Alison O'Daffer, Joyce Yi-Frazier, Abby R Rosenberg

**Affiliations:** 1 Palliative Care and Resilience Lab Center for Clinical and Translational Research Seattle Children’s Research Institute Seattle, WA United States; 2 Department of Psychiatry and Behavioral Sciences University of Washington School of Medicine Seattle, WA United States; 3 Cambia Palliative Care Center of Excellence University of Washington Seattle, WA United States; 4 Department of Pediatrics University of Washington School of Medicine, WA United States

**Keywords:** telemedicine, smartphone, mobile phones, mHealth, mobile apps, health services

## Abstract

Mobile health (mHealth) technologies have provided an innovative platform for the deployment of health care diagnostics, symptom monitoring, and prevention and intervention programs. Such health-related smartphone apps are universally accepted by patients and providers with over 50 million users worldwide. Despite the rise in popularity and accessibility among consumers, the evidence base in support of health-related apps has fallen well behind the rapid pace of industry development. To bridge this evidence gap, researchers are beginning to consider how to best apply evidence-based research standards to the systematic synthesis of the mHealth consumer market. In this viewpoint, we argue for the adoption of a “hybrid model” that combines a traditional systematic review with a systematic search of mobile app download platforms for health sciences researchers interested in synthesizing the state of the science of consumer apps. This approach, which we have successfully executed in a recent review, maximizes the benefits of traditional and novel approaches to address the essential question of whether popular consumer mHealth apps work.

## Introduction

In the past decade, smartphones have become ubiquitous across personal, social, and vocational domains [1], regardless of gender, race, ethnicity, and socioeconomic status [2]. There are 3.5 billion smartphone users worldwide [3]. Nearly 75% of Americans own a smartphone and 83% of smartphone owners never leave home without it [1,4]. Mobile health (mHealth) technologies may improve access to health care by overcoming financial constraints and geographical barriers; 73% of families living below the poverty line have 1 or more smartphones even if they lack access to other resources, and telehealth enables expanded access to services in rural communities [5,6]. Over 50 million people use apps for health monitoring and diagnostic purposes worldwide [7]. Smartphone app-based tools for diagnosis, symptom monitoring, behavioral change, provider–patient communication, and disease-related education have become increasingly popular and have the potential to improve health and behavioral outcomes [8-10].

Previous research suggests that both patients and providers have a strong interest in utilizing mHealth technologies as part of health care practices, particularly apps that are supported by research evidence [11-13]. However, the research evidence lags far behind the exponential growth of publicly available apps for consumer download. As of 2020, there were approximately 101,000 mHealth apps available in major app stores with 3.7 billion annual downloads, and the market is forecasted to reach US $312 billion by 2027 [14-17]. This presents a significant research–practice divide with the widespread adoption of app-based health care tools and interventions that may not be backed by science [18].

## Viewpoint Structure

This viewpoint is structured according to the main points of the “design science” framework for information systems research: problem identification, objectives, design and development, and demonstration [19].

### Problem

Further fracturing the research–practice divide into a chasm, there are no gold-standard methods for evaluating the evidence in support of the efficacy of publicly available consumer apps within the growing mHealth industry.

### Objectives

To address the research–practice chasm, we endeavored to develop methodologically rigorous and reproducible standards for evaluating whether publicly available mHealth tools and interventions work. In this viewpoint, our primary objective is to provide a narrative description of the lessons we learned from the process of designing a recent study evaluating the evidence in support of popular stress management and psychosocial wellness apps [20].

### Design and Development

We considered methodological approaches such as a traditional systematic review and a novel search of mobile app download platforms. A systematic review is particularly well-suited for research questions pertaining to feasibility and efficacy of apps developed in research settings. Novel searches of mobile app download platforms allow researchers to examine the functionality and usability of popular apps. Neither was sufficient alone for novel research questions pertaining to the state of the science of popular apps available for consumer download. Ultimately, we decided on a “hybrid model” combining a systematic review with a systematic search of mobile app download platforms, a methodological approach that was “not too hot, not too cold, but just right.” We recommend that researchers interested in the review and synthesis of publicly available consumer mHealth apps in their respective disciplines utilize a “hybrid model” such as this to guide research design conceptualization.

### Demonstration

We present 2 illustrative examples of studies following a “hybrid model” design, while providing additional citations of other successful studies.

### Porridge Bowl #1: Systematic Review

First, we explored the possibility of utilizing a systematic review to synthesize the consumer app landscape. This traditional approach benefits from gold standards that have been extensively detailed in references such as the *Cochrane Handbook for Systematic Reviews of Interventions* [21] which covers all aspects of review planning from idea inception to data collection and analysis. Health science researchers interested in summarizing the state of the science in mHealth-related topics have conducted traditional systematic reviews utilizing databases of references such as Ovid MEDLINE, Embase, Cochrane Central Register of Controlled Trials, Web of Science, Scopus, and PsycINFO using the PICO framework to inform the search, where prespecified parameters include Patient problem/Population, Intervention, Comparator, and Outcomes of interest [22-31]. Previous smartphone app systematic reviews have spanned topics such as health behavior change interventions, medication management, and cognitive behavioral therapy and behavioral activation apps for depression [22-30].

Despite the advantage of rigorous well-defined methods with reproducible results, there are fatal flaws in the application of this approach specifically for those interested in asking and answering research questions pertaining to the state of the science of the mHealth industry space. First, the majority of apps developed in traditional laboratory-based and research settings are not available for public download and require private access codes provided to research participants only [26]. Second, less than 1% of mHealth apps across a range of previously explored health domains had corresponding scientific publications describing their efficacy [20,24,32]. Taken together, there is little to no overlap between apps evaluated in traditional systematic reviews (which query the existing literature in extensive library databases) and the types of consumer apps available for public download (which query the existing apps available for your mobile devices). For example, a systematic review of mHealth psychological interventions for anxiety which showed small to medium effect sizes can only tell us about the efficacy of apps that have been formally tested in clinical trials [26]; it does not provide information regarding the efficacy of the types of apps we all download directly onto our smartphones based on popularity metrics such as Top 100 lists, Editor’s Picks, media buzz, and consumer ratings. Apps developed in industry and research settings are siloed tracks. Thus, conclusions drawn from traditional systematic reviews are limited for informing the types of novel research questions endeavoring to synthesize the landscape of 101,000+ mHealth apps currently available to smartphone users.

### Porridge Bowl #2: Systematic Search of Mobile App Platforms

Second, we explored the possibility of utilizing a systematic search of mobile app platforms to synthesize the state of the science of the mHealth industry. In the past decade, mHealth researchers have conducted mobile app download platform searches as an alternative method to traditional systematic reviews befitting the consumer app space [23,33-40]. (Editorial note: Note that JMIR journals do not apply the term "systematic review" to these kinds of studies, but calls them "Systematic Searches on App Stores" or similar; the term "systematic review" is reserved for literature reviews. Other publishers/journals may not distinguish these different study types). This user-centered approach prioritizes broad applicability of findings to day-to-day mobile phone users seeking digital health-management tools and interventions. Mobile app download platforms are utilized as the equivalent of library databases for data extraction in order to identify, screen, and review apps for inclusion and exclusion. In addition, the Mobile Application Rating Scale (MARS) is a commonly used tool for assessing the quality of mHealth apps and provides objective classifications (eg, price, platform, aspects of health targeted), and subjective subscale ratings in the domains of engagement, functionality, aesthetics, and information quality along with a composite app quality rating [41]. The 23-item scale has demonstrated high internal consistency and fair interrater reliability with independent coders [41]. Thus, researchers may compare and gauge the potential impact or value of mHealth programs that happen to be available for consumer download. In the United States, 54.4% of smartphone owners use Android devices and 44.3% use Apple devices [42]. Thus, the majority of existing systematic app searches (and thus app content and quality assessments) span Apple and Android platforms only [33,34,38-40]. Previous systematic searches of mobile app download platforms have spanned a wide range of topics such as smoking cessation, mindfulness, physical activity promotion, and pharmacology education [33,35,36,38,40].

Recent methodological innovations are as follows: (1) The European Innovation and Knowledge mHealth Hub is a project established in 2020 by the International Telecommunication Union (ITU) in collaboration with the World Health Organization (WHO) [43]. The mHealth Hub offers an overview of 24 health app assessment frameworks evaluating domains including privacy, transparency, safety, and technical stability; this provides additional resources and tools for researchers to systematically synthesize app features and content. (2) Big data innovations have focused on developing automated methods to extract information on app features and components from the web using natural language processing and text analytics [44].

Despite the advantages of “real-world” representativeness of apps, research questions are limited to a synthesis of user-centered metrics such as mHealth app usability, functionality, engagement, consumer appeal, and content [45-47]. Although such scientific inquiries are important, a systematic search of mobile app platforms does not provide information on whether and how an app works, which are the scientific merits that providers and researchers rely on for establishing evidence-based standards of care and treatment recommendations. In addition, FDA-approved apps that are validated medical devices “for diagnosis of disease or other conditions, or the cure, mitigation, treatment, or prevention of a disease” are not separately listed from nonvalidated health apps; this further limits the ability of users to make informed choices about which health apps are certified tools subject to regulatory oversight [48,49].

### Porridge Bowl #3: “Just Right” Hybrid Designs

**Figure 1:**
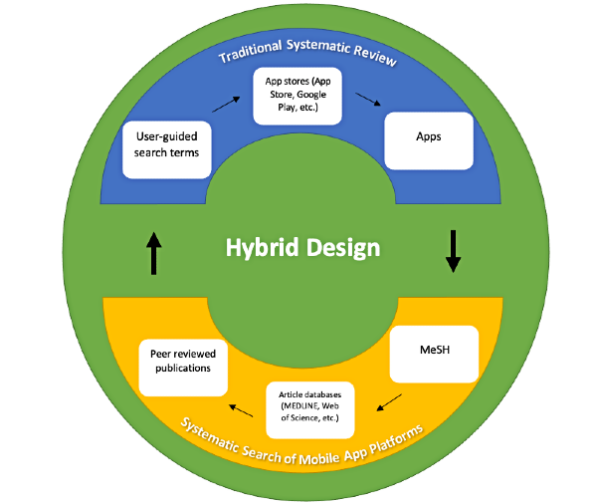
Hybrid design model.

To recap, systematic review methodology confers the benefit of providing information on the scientific merit of apps developed in research settings but do not represent the “real-world” consumer apps that we all download to our smartphones. App download platform search methodology, by contrast, confers the benefit of “real-world” app quality, content, and representation but not of scientific merit. Recent research has utilized hybrid design methodology by combining traditional literature review methods with systematic searches of mobile app platforms, bridging well-established traditional and novel methodologies (Figure 1) [20,24,25,32]. Next, we describe 2 illustrative examples.

### Illustrative Example #1: Systematic App Search Followed by Literature Review

We utilized a hybrid design to evaluate the following research aims: (1) What are the therapeutic contents and features of popular stress management and psychosocial wellness apps, and (2) Which apps, if any, are supported by peer-reviewed original research publications? This study was conducted in 3 stages [20].

#### Step 1 (User-Centered Approach)

We identified conventional self-help search terms from the background literature and refined the list in consensus conversations among our interdisciplinary team of intervention science researchers, health services researchers, physicians, social workers, and psychologists. Then, we input search terms directly into Android and Apple iOS mobile app search engines to identify consumer apps meeting inclusion criteria. Two authors (NL and AO) independently reviewed all apps. Independent raters created a comprehensive database with content categories representing all intervention ingredients identified, and abstracted relevant data from product pages.

#### Step 2 (Traditional Review Approach)

A literature review was conducted via Google Scholar, Medline, and PsycINFO databases of all commercially available apps identified in Step 1 using the search terms “[app name]” AND smartphone. Data on feasibility and efficacy outcomes were abstracted from the journal articles, and risk of bias was coded by 2 independent raters (NL and AO).

#### Step 3 (Synthesize Findings)

Using this hybrid design, we answered these complementary questions relevant to the state of the science of the mHealth market: Which everyday consumer apps are popular and what is their treatment content? What is the evidence in support of everyday consumer apps available to the general public?

### Illustrative Example #2: Cyclical Approach

de la Vega and colleagues [32] conducted a systematic review of pain-related apps for pain symptom assessment and education. Utilizing a cyclical model, they conducted independent parallel searches, in one case starting with a systematic search of mobile app download platforms followed by a traditional literature review, and in the other case starting with a traditional literature review followed by a systematic search of mobile app download platforms.

#### Search 1/Step 1 (Traditional Review Approach)

A literature review was conducted across 17 relevant scientific databases (Medline, PsycINFO, Web of Science, etc.) utilizing Boolean-operator pain “AND” mobile app search terms.

#### Search 1/Step 2 (User-Centered Approach)

Apps meeting inclusion criteria identified in Search 1/Step 1 were input directly into 5 mobile app download platforms (Apple Store, Google Play, Blackberry App World, Nokia Store, and Windows Play Store) to determine if research-supported apps were available for public download.

#### Search 2/Step 1 (User-Centered Approach)

User-friendly search terms related to pain management were input directly into mobile app download platforms (Apple Store, Google Play, Blackberry App World, Nokia Store, and Windows Play Store).

#### Search 2/Step 2 (Traditional Review Approach)

A literature review was conducted via the aforementioned library databases of all commercially available apps identified in Search 2/Step 1 by app name. Authors also searched Google and MyHealthApps.net to learn about app origins and creators.

#### Step 3 (Synthesize Findings)

Using this hybrid design, de la Vega and colleagues [32] answered these complementary questions relevant to the state of the science of the mHealth market: Which research-supported apps identified for inclusion via a traditional literature review are commercially available? How many commercially available apps identified for inclusion via a systematic search of app download platforms are supported by peer-reviewed publications?

Strengths of the “hybrid model” included the cumulative advantages of systematic review and systematic search methodologies, and a comprehensive holistic analysis of the subset of “real-world” consumer apps that are research based. A hybrid design was the only approach that would allow researchers, clinicians, and patients/consumers alike to answer the essential question of whether consumer app-based health care tools and interventions that have been increasingly adopted worldwide actually work and what therapeutic content and features are incorporated in their design. In our previous study, we found further evidence to support the conclusion that apps developed in industry and research settings are siloed (ie, consumer apps developed in the mHealth industry and available for public download rarely have corresponding research publications). The “hybrid” approach allows health sciences researchers to identify the subset of apps that are both research based and publicly available despite the fact that there is no existing database/repository for consumers seeking evidence-based care.

## Discussion

Smartphone apps to address a diverse array of health care needs are being developed at a rapid rate and are widely adopted worldwide. However, the scientific merit of “real-world” apps remains largely understudied and unknown. This is due, in part, to the absence of well-established methods for the evaluation of the efficacy of consumer apps. Of the 2 more common approaches to evaluation, neither is “just right” to determine both evidence base and quality for the consumer app space. Traditional literature reviews are valuable for gathering and synthesizing information regarding the scientific backing of popular apps in the form of feasibility and efficacy study data. Review of mobile app search engines allows for a direct synthesis of popular consumer apps and user-centered metrics such as usability, engagement, functionality, and app content. The “hybrid model” described in this viewpoint allows researchers to address novel research questions leveraging the complementary strengths of a systematic review and app search engine review. Although not without its limitations, hybrid approaches provide a unique opportunity to develop and iteratively refine methodologies for synthesizing the state of the science of the quickly evolving consumer mHealth market. Future research should focus on the standardization of mobile app download platform searches and systematic blending of traditional and novel methodological approaches via “just-right” hybrid designs. Such research endeavors will help bridge the research–practice chasm by rigorously evaluating digital health industry solutions to health care problems. Ultimately, this will help us understand whether popular apps work and inform mHealth clinical practice guidelines.

## Abbreviations

ITU: International Telecommunication Union
MARS: Mobile Application Rating Scale
mHealth: mobile health
WHO: World Health Organization
